# Development of Asialoglycoprotein Receptor-Targeted Nanoparticles for Selective Delivery of Gemcitabine to Hepatocellular Carcinoma

**DOI:** 10.3390/molecules24244566

**Published:** 2019-12-13

**Authors:** Anroop B. Nair, Jigar Shah, Bandar E. Al-Dhubiab, Snehal S. Patel, Mohamed A. Morsy, Vimal Patel, Vishal Chavda, Shery Jacob, Nagaraja Sreeharsha, Pottathil Shinu, Mahesh Attimarad, Katharigatta N. Venugopala

**Affiliations:** 1 Department of Pharmaceutical Sciences, College of Clinical Pharmacy, King Faisal University, Al-Ahsa 31982, Saudi Arabia; baldhubiab@kfu.edu.sa (B.E.A.-D.); momorsy@kfu.edu.sa (M.A.M.); sharsha@kfu.edu.sa (N.S.); mattimarad@kfu.edu.sa (M.A.); kvenugopala@kfu.edu.sa (K.N.V.); 2 Department of Pharmaceutics, Institute of Pharmacy, Nirma University, Ahmedabad 382481, Gujarat, India; jigsh12@gmail.com (J.S.); email2vimal.patel@gmail.com (V.P.); 3 Department of Pharmacology, Institute of Pharmacy, Nirma University, Ahmedabad 382481, Gujarat, India; snehalpharma53@gmail.com (S.S.P.); chavdavishal2@gmail.com (V.C.); 4 Department of Pharmacology, Faculty of Medicine, Minia University, El-Minia 61511, Egypt; 5 Department of Pharmaceutical Sciences, College of Pharmacy, Gulf Medical University, Ajman 4184, UAE; sheryjacob6876@gmail.com; 6 Department of Biomedical Sciences, College of Clinical Pharmacy, King Faisal University, Al-Ahsa 31982, Saudi Arabia; spottathail@kfu.edu.sa; 7 Department of Biotechnology and Food Technology, Durban University of Technology, Durban 4000, South Africa

**Keywords:** carrier, targeted delivery, gemcitabine, clearance, organ distribution

## Abstract

Selective targeting of anticancer drugs to the tumor site is beneficial in the pharmacotherapy of hepatocellular carcinoma (HCC). This study evaluated the prospective of galactosylated chitosan nanoparticles as a liver-specific carrier to improve the therapeutic efficacy of gemcitabine in HCC by targeting asialoglycoprotein receptors expressed on hepatocytes. Nanoparticles were formulated (G1–G5) by an ionic gelation method and evaluated for various physicochemical characteristics. Targeting efficacy of formulation G4 was evaluated in rats. Physicochemical characteristics exhibited by nanoparticles were optimal for administering and targeting gemcitabine effectively to the liver. The biphasic release behavior observed with G4 can provide higher drug concentration and extend the pharmacotherapy in the liver target site. Rapid plasma clearance of gemcitabine (70% in 30 min) from G4 was noticed in rats with HCC as compared to pure drug (*p* < 0.05). Higher uptake of gemcitabine predominantly by HCC (64% of administered dose; *p* < 0.0001) demonstrated excellent liver targeting by G4, while mitigating systemic toxicity. Morphological, biochemical, and histopathological examination as well as blood levels of the tumor marker, alpha-fetoprotein, in rats confirmed the curative effect of G4. In conclusion, this study demonstrated site-specific delivery and enhanced in vivo anti-HCC efficacy of gemcitabine by G4, which could function as promising carrier in hepatoma.

## 1. Introduction

Hepatocellular carcinoma (HCC) is one of the predominant forms of liver cancer and accounts approximately for 90% of total hepatic malignancy. Systemic chemotherapy is preferred in patients with advanced HCC [1]. Conventional chemotherapy is less effective in treating HCC essentially due to the poor specificity of these agents to malignant tumor cells. In addition, the nonselectivity of chemotherapeutic drugs frequently causes severe toxic side effects that often limit dose escalation, undesirable biodistribution, poor response, rapid clearance, quick disease relapse, and eventually drug resistance [2]. Thus, developing new treatment paradigms to provide optimal treatment for HCC has become a fascinating topic in drug delivery research. Current therapeutic strategies in HCC aim to improve the clinical potential of chemotherapeutic agents by targeting them to the tumor site [3]. Various targeting approaches have been explored to improve the pharmacotherapy of cytotoxic agents by delivering them to the tumor cells [4]. Among these, the active targeting of anticancer drugs by developing carrier systems with specific ligands has attained much attention [5]. Typically, these nano-sized ligand-bound systems will potentially transport the drug to the tumor site and bind effectively to the overexpressed target receptors in cancer cells. The prospective of lipid and polymer-based carriers coupled with ligands has been extensively explored during the last few decades and has found some encouraging results [6,7]. Lipid-based formulations such as liposomes, micelles, and emulsions with diverse ligand moieties to target liver cells have been developed [8]. Alternatively, proteins, peptides, and nucleic acids have also been explored to target liver cancer. On the other hand, polymeric nanocarriers have also demonstrated certain progress in targeting cancer cells in absence of ligands due to their higher permeability to the tumor vasculature, along with an enhanced permeability and retention effect [9,10]. Nevertheless, the pharmacological and therapeutic responses are improved when these carriers are combined with ligands [5,11].

Gemcitabine, a pyrimidine nucleoside antimetabolite, has an established and significant role in the pharmacotherapy of diverse types of human cancers such as pancreatic, lung, breast, bladder, ovarian, and neck [12]. A combination therapy of gemcitabine with paclitaxel is found to be effective as a first-line therapy in patients with metastatic breast cancer [13]. Similarly, gemcitabine is also combined with cisplatin and is a primary therapy for patients with advanced or metastatic non-small cell lung cancer [14]. In addition, gemcitabine shows a promising effect and is used as a first-line therapy in advanced or metastatic adenocarcinoma of the pancreas [15]. Several clinical studies shown notable results in HCC treatment when gemcitabine was combined with other anticancer agents, including sorafenib, oxaliplatin, carboplatin, and bevacizumab [3]. Unfortunately, monotherapy of gemcitabine shows moderate antitumor activity in patients with advanced HCC, though it exhibits good antitumor effects in human hepatoma cell line [16,17]. Moreover, polytherapy regimens of gemcitabine have demonstrated notable outcomes during the treatment of advanced HCC [18,19]. The data from clinical trials also suggests relatively low toxicity issues of gemcitabine, but the adverse effects related to myelosuppression and pulmonary toxicity are still a concern [20]. From this perspective, developing a nanocarrier system with particular ligands that can recognize and interact with specific target surface receptors in HCC cells can provide selective targeting of gemcitabine. The upregulation of these receptors can ensure maximum therapeutic efficacy and could be beneficial in the pharmacotherapy of HCC.

Chitosan (poly-β-1,4-glucosamine) is a natural linear polysaccharide made by deacetylation of chitin (poly-β-(1→4)-*N*-acetyl-d-glucosamine) and has molecular mass in the range of 10 to over 1000 kDa [21]. It is nontoxic, biocompatible, and biodegradable polymer, extensively used in pharmaceutical carriers [22]. Chemical modification of chitosan can yield galactosylated chitosan. The modification of chitosan is usually carried out through the primary amine group of the polymer, which can conjugate with lactobionic acid. This derivative contains galactose molecules, which are active ligands with specific liver targeting function. The prospective of galactosylated chitosan has been exploited as a liver-specific carrier to enhance the therapeutic efficacy of various drugs [23,24,25]. 

Asialoglycoprotein is a heterooligomer with molecular mass of 41 kD and is composed of two homologous polypeptides. This polypeptide is expressed in the liver and is minimally present in the other human tissues [26]. This transmembrane protein is a surface receptor situated on the hepatocyte membrane towards the sinusoids and specifically can recognize the terminals of β-d-galactose [27]. Indeed, each hepatocyte contains about 500,000 asialoglycoproteins and has specificity for glycoproteins having galactose groups. This endogenous glycoprotein is over expressed in HCC and has high binding capacity and allows efficient cellular uptake of galactose ligands. Although it is abundantly expressed on hepatocytes, the asialoglycoprotein receptors can clearly recognize and bind the galactose ligands located on the external surface of nanoparticles. Hence, the objective of the current investigation was to prepare gemcitabine-loaded nanoparticles utilizing galactosylated chitosan and evaluate its prospective for hepatocyte-specific delivery in HCC. Five gemcitabine-loaded galactosylated chitosan nanoparticles were prepared (G1–G5) and assessed for various physicochemical properties. Formulation G4 was further studied for in vitro drug release, in vivo blood disappearance, and organ distribution. Biochemical and histological analyses were performed to investigate the curative effect of G4 in HCC rat model.

## 2. Result and Discussion

### 2.1. Synthesis of Galactosylated Chitosan

The amide bond formation between the primary amine group of chitosan and carboxyl group of lactobionic acid was carried out using 1-ethyl-3-(3-dimethyl aminopropyl) carbodiimide and *N*-hydroxylsuccinimide, which are widely used as chemical cross linking agents. The choice of 1-ethyl-3-(3-dimethyl aminopropyl) carbodiimide is because of its water solubility. N-Hydroxylsuccinimide was included to guard the sensitive intermediate (*O*-acylurea) and decrease its conversion into N-acylurea, as well as to avoid the racemization of the final product [28]. The (N,N,N′,N′-tetramethylethylenediamine/HCl buffer system was used to promote the reaction. The chemical structure of galactosylated chitosan was established by FTIR spectra, which show peaks at 1610 cm^−1^ and 1595 cm^−1^, signifying the formation of an amide bond.

### 2.2. Preparation of Gemcitabine Nanoparticles

The clinical efficacy of several anticancer drugs against HCC is primarily limited because of their inability to deliver therapeutically relevant drug levels to the target site. Active targeting of anticancer drugs using nanoparticles has been demonstrated as a promising approach that could change the biological behavior and body distribution [5]. However, the characteristics of such nanoparticles should be optimal to attain the desired therapeutic effect. In the current study, five gemcitabine-loaded galactosylated chitosan nanoparticles were prepared (G1–G5) by an ionic gelation technique with varying proportions of drug and polymer weight, as seen in Table 1.

### 2.3. Characterization of Gemcitabine Nanoparticles

Physicochemical characteristics of nanoparticles, particularly particle size, particle shape, and surface charge, have significant effect in cellular uptake and are therefore crucial for drug targeting [29]. The physicochemical characteristics of prepared nanoparticles are summarized in Table 1. It is apparent from Table 1 that the percentage yield (approximately 70–75%) of nanoparticles (G1–G5) were high and reproducible, which also substantiates the suitability of the adopted method. Drug entrapment is a key parameter in nanoformulations in order to achieve greater therapeutic efficiency of the encapsulated drugs [30]. The EE of drug is mainly dictated by the partition coefficient of the drug and its inherent solubility in the specific polymer. However, the solubility of drug in polymer is normally influenced by the physicochemical properties (molecular weight and chemical nature of drug and polymer) as well as the probable drug–polymer interaction [31]. The effect of polymer concentration (40–200 mg) on the %EE and %DL values were assessed and are depicted in Table 1. The results indicate that an increase in polymer content in the formulation resulted in an increase in the %EE (54–72%) and reached the highest when the drug:polymer ratio was 1:8 (formulation G4). However, the variation in %EE observed in prepared nanoparticles suggests that the polymer concentration in the formulation can play a vital role in %EE. The moderately high %EE results observed (54–72%) could be attributed to the physicochemical characteristics of the drug as well as the polymer, which in turn might have favored the drug entrapment as mentioned before. As anticipated, the %DL (6–18%) values of prepared nanoparticles decreased with the increase in polymer content, as seen in Table 1, in the present experimental conditions. Drug content is equally important as it symbolizes the uniformity of active constituents in finished drug products. The gemcitabine concentrations in the prepared formulations were high (>90%) and were comparable, as seen in Table 1. The low standard deviation (1.56–2.94%) values observed signify uniformity of drug content in the different formulations. Overall, the data suggests that the change in drug:polymer ratio has no influence on the drug content in the present experimental settings.

**Figure 1:**
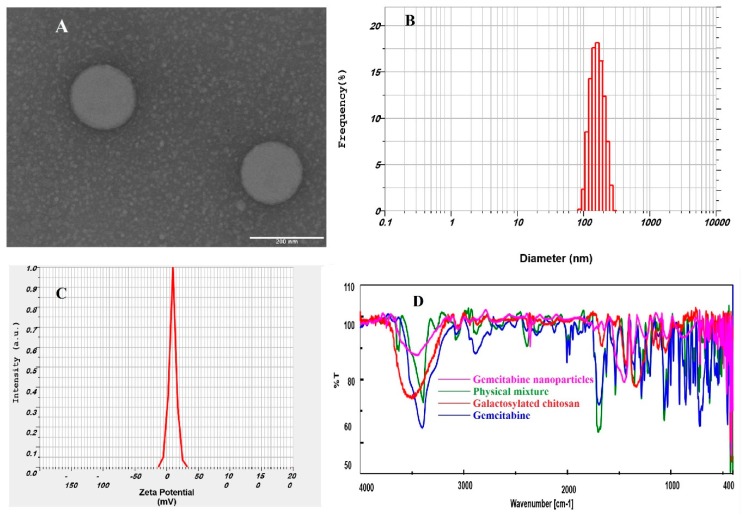
Characteristics of gemcitabine-loaded nanoparticles (G4). (**A**) Transmission electron microscopy image; (**B**) size distribution histogram; (**C**) zeta potential distribution; and (**D**) Fourier transform infrared spectroscopy.

Prepared nanoparticles were further assessed for particle size, size distribution, zeta potential, and polydispersity index. Particle size is a key parameter of carriers as it modulates the delivery and targeting of drug molecules. Nanoparticles having particle sizes exceeding a certain diameter (>250 nm) seldom infiltrate liver parenchymal cells, while smaller nanoparticles have greater capacity to accumulate at the hepatic tumor region [32,33]. On the contrary, to establish efficient endocytosis, nanoparticles should be sufficiently large to avoid rapid systemic clearance or to evade engulfment by macrophages located in the reticuloendothelial system [32,33]. The TEM image, seen in Figure 1A, shows that the nanoparticles were spherical in shape, having uniform surfaces without any aggregation, and were nano-sized. Particle size analysis reveals that the size of particles had an approximate Gaussian distribution, unimodal, and relatively narrow particle size distribution, as seen in Figure 1B. Thus, the prepared formulations have the desired characteristics suitable for targeting parenchymal cells of the liver.

Zeta potential is another important index, which reveals the intensity of repulsion between neighboring similarly charged particles and provides insight into the probability of particle aggregation and physical stability [34]. A representative image of zeta potential values for the prepared nanoparticles is presented in Figure 1C. Data in Table 1 show that the zeta potential values of the developed nanoparticles did not change between the formulations since their compositions were similar. The measured positive zeta potential values (19 to 22 mV) add moderate repulsion between the vesicles and electrostatic stabilization to provide a stable dispersion. The positive zeta potential observed here could be due to the donation of positive charge furnished by the amino groups present in the chitosan units [33]. In addition, the positive charge observed in the nanoparticles could be beneficial for delivering the encapsulated drug because of possible interactions between the positively charged nanoparticles surface and oppositely charged cell membranes [33]. Polydispersity index is mainly determined to assess the homogeneity of prepared nanoparticles. The polydispersity index values of prepared particles ranged from 0.22–0.30, confirming its narrow particle size distributions. Overall, the physicochemical properties of the prepared nanoparticles (G1–G5) did not vary significantly; however, formulation G4 was selected as a representative for further studies as it showed the highest yield, EE, and drug content.

### 2.4. FTIR

The FTIR spectra of gemcitabine, galactosylated chitosan, the physical mixture, and gemcitabine-loaded nanoparticles are displayed in Figure 1D. The infrared spectrum of gemcitabine HCl showed distinct sharp peaks at 3450, 2930, 1740, 1540, 1176, and 724 cm^−1^ because of the existence of N–H stretching of amines, alkenes or aliphatic C–H, C=O, aromatic C=C, and C–N amines, and aromatic C–H, respectively. All these peaks were also observed in the physical mixture. However, the corresponding drug peaks either disappeared or were reduced in intensity in the infrared spectra of drug-loaded nanoparticles, which indicates incorporation of gemcitabine in the nanoparticles. The spectra of galactosylated chitosan and drug-loaded nanoparticles did not present any significant variation, suggesting there is no incompatibility between the drug and polymer, as described in the literature [35].

### 2.5. DSC

**Figure 2:**
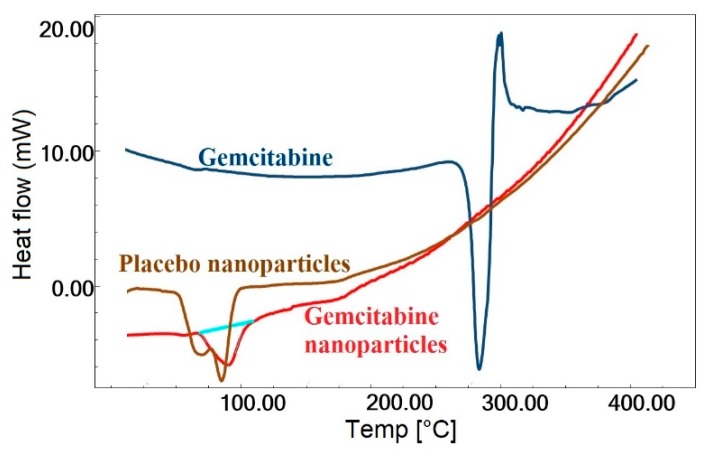
The differential scanning calorimetry thermographs of gemcitabine, placebo nanoparticles, and gemcitabine-loaded nanoparticles.

Differential scanning calorimetry (DSC) was used to characterize the state of gemcitabine as well as the changes in thermodynamic properties that might have happened inside the nanoparticles. Changes in processes such as melting and solid phase transformations were observed as indicated by endothermic or exothermic peaks. A sharp endothermic peak characterizing the melting point of gemcitabine HCl was observed at 287.3 °C, as seen in Figure 2, indicating its crystalline character. The diffraction pattern of the placebo nanoparticles revealed a broad endothermic peak at 88.5 °C, which could be attributed to the glass transition temperature of galactosylated chitosan [36]. A similar thermogram was observed in nanoparticles containing gemcitabine (G4), but the endotherm was slightly shifted to 92.8 °C, probably due to inclusion of the drug with the polymer. However, no apparent endothermic melting peak of the drug was observed in nanoparticles containing gemcitabine. The existence of noncrystalline gemcitabine within the nanoparticles was indicated by the absence of an endothermic melting peak of the drug in the DSC thermogram.

### 2.6. Drug Release

**Figure 3:**
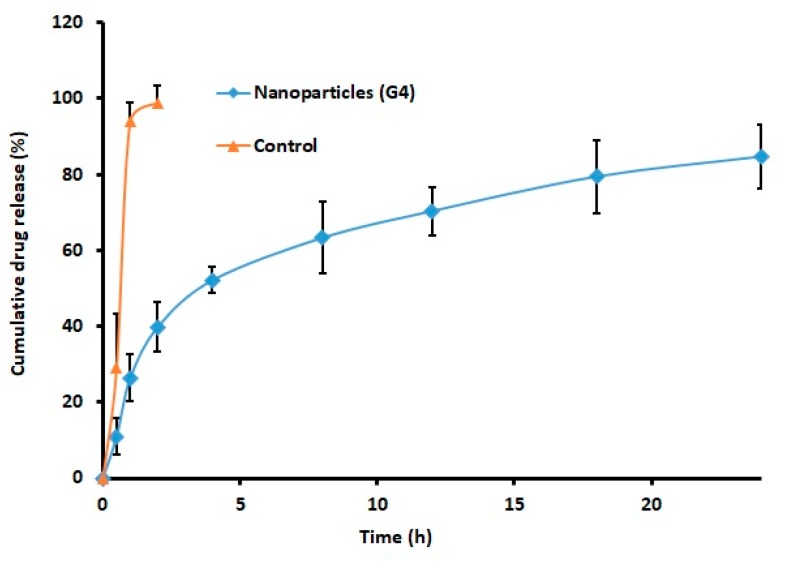
Comparison of the cumulative percentage of gemcitabine released from nanoparticles (G4) and pure drug (control). The in vitro drug release study was performed in a dialysis tube using phosphate-buffered saline as the release medium. The data represents the average ± SD of six trials.

Drug release from the nanoparticles is a prerequisite for its therapeutic effect. The analysis of drug release from the formulation can provide adequate indication about its real time in vivo behavior. Figure 3 illustrates the cumulative percentage gemcitabine released from G4 and control at specified time period. It is clear from Figure 3 that the in vitro release profile of gemcitabine nanoparticles was biphasic. A greater amount of drug (>50%) was released in the initial stage (4 h), which was presumably due to the diffusion of gemcitabine from the nanoparticle surface or shallow layer in the solution. Thereafter, the drug release was markedly restrained, occurred at a slow pace, and was steadily increased and continued up to 24 h. The slow and sustained release occurred in the steady release stage (4–24 h) may be attributed to the drug diffusion through the polymeric core as well as the matrix degradation, which is a slow process [19]. The profile seen in Figure 3 is certainly incomplete, as the percentage of gemcitabine released was approximately 85% in 24 h. In contrast, pure drug exhibited rapid and complete release in 2 h. Overall, the data indicates the capability of the prepared nanoparticles to provide sustained and prolonged release of gemcitabine. Indeed, the steady release of gemcitabine from the carrier within the target site is advantageous since it would bathe the organ for a long time. 

The release mechanism for G4 was evaluated using various mathematical release kinetic models. The data of sum of square of residuals (SSR) were calculated as 1610.08, 876.18, 410.33, 502.96, 115.46, and 1079.12 for zero order, first order, Higuchi, Korsmeyer–Peppas, Weibull, and Hixon–Crowell model, respectively. The release pattern of gemcitabine from G4 was fitted into a Weibull model, exhibiting a high r^2^ value (0.9609), low SSR value (115.46), and F value (16.49). Therefore, the release of gemcitabine from G4 followed a Weibull diffusion-controlled mechanism. Stability study results did not exhibit any major variation in particle size, drug content, or release, suggesting the product is quite stable at room temperature.

### 2.7. Blood Disappearance and Organ Distribution

**Figure 4:**
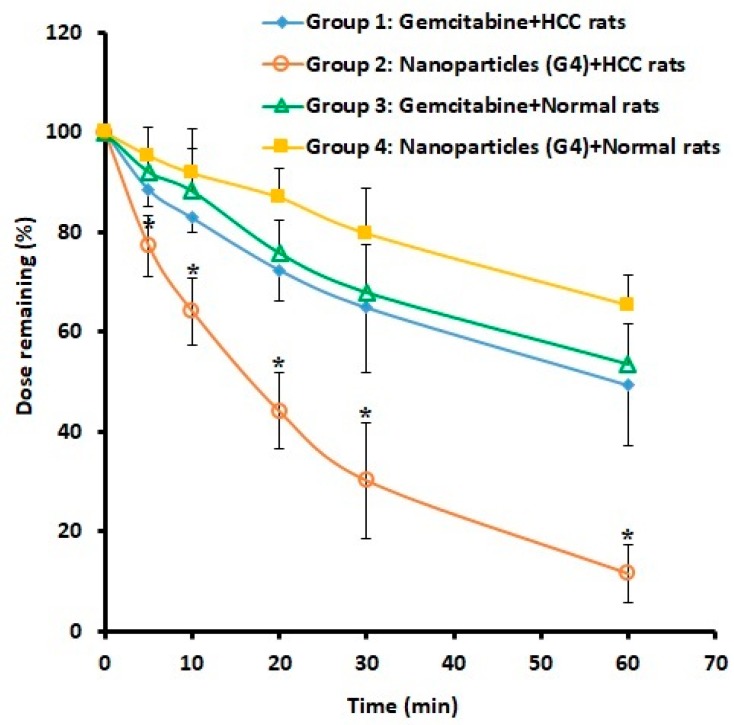
Comparison of drug plasma profiles in rats after administration of gemcitabine either free or in combination with nanoparticles (G4) in various treatments. The profile of group 2 is statistically (*) different (*p* < 0.05) when compared to other treatments (groups 1, 3, and 4). Group 1: HCC + Gemcitabine. Group 2: HCC + Nanoparticles. Group 3: Normal + Gemcitabine. Group 4: Normal + Nanoparticles. The values represent the average ± SD (*n* = 6). HCC: hepatocellular carcinoma.

Targeted drug delivery systems are expected to provide selective delivery of drug molecules and thereby maximize the therapeutic efficacy while minimizing the chance of adverse effects. In HCC, the asialoglycoprotein receptor present on the membrane of hepatocytes is highly expressed. Hence, the ability of selected nanoparticles (G4) with ligand (galactose) to provide selective targeting of HCC was evaluated by assessing blood disappearance and organ distribution. Groups 1 and 2 of rats were induced with HCC and groups 3 and 4 had normal rats. Drug and nanoparticles were administered intravenously at a dose of 1 g/m^2^. Gemcitabine plasma disappearance was monitored for 60 min and subsequently the drug content in organs were determined. Figure 4 shows the percentage of gemcitabine present in plasma in various treatments. It is clear from Figure 4 that the plasma concentration–time curve of group 2 (HCC rats administered with nanoparticles) was significantly different (*p* < 0.05) in comparison to the other treatments (groups 1, 3, and 4). The clearance of gemcitabine from systemic circulation seems to be very rapid in group 2 and was reduced to about 44% and 30% 20 and 30 min after administration, respectively. On the contrary, the plasma drug concentration plot of gemcitabine-alone administered groups (1 and 3) were similar and statistically insignificant. In both groups (1 and 3), approximately 50% of administered gemcitabine was found in the plasma at 60 min. The reason for the reduction in drug level in these groups (1 and 3) could be related to the short half-life of gemcitabine (32 to 94 min). In the case of group 4 (normal rats administered with nanoparticles), the decline in plasma drug level was gradual and moderately higher than in groups 1 and 3 (although statistically insignificant) and approximately 65% of the injected gemcitabine was found after 60 min. The possible reason for the gradual decline could be that the drug molecules are entrapped inside the nanoparticles and were not released for metabolism and elimination. It is also evident from Figure 4 that the rapid clearance of gemcitabine from nanoparticles happens only in diseased animals (group 2), while not in group 4 (normal rats). Assessment of the biodistribution of the drug in different organs is important for determining the destiny of gemcitabine that has disappeared from the plasma.

**Figure 5:**
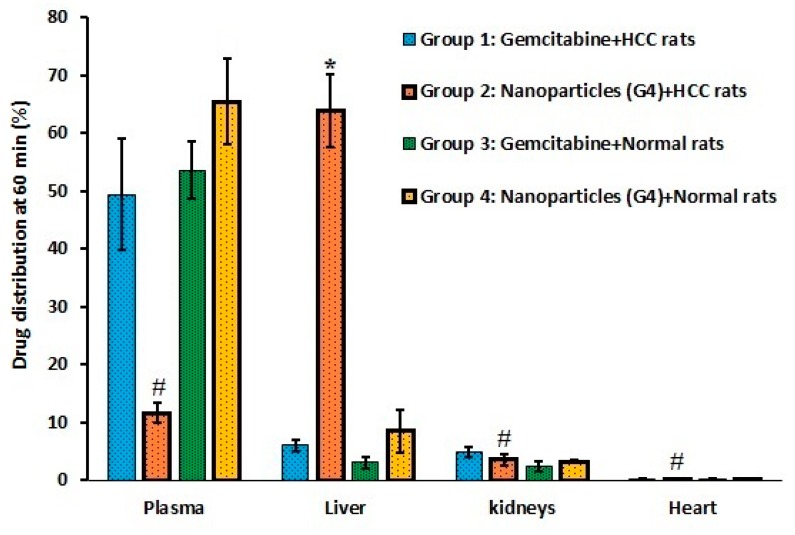
Comparison of organ and plasma distribution in rats 60 min after intravenous administration of gemcitabine either free or in combination with nanoparticles (G4) in various treatments. *Significant difference (*p* < 0.0001) in gemcitabine amount in liver tissues of group 2 compared to amount in liver tissues of other treatments (groups 1, 3, and 4). #Significant difference (*p* < 0.0001) of gemcitabine amount in liver of group 2 compared to the amount in group 2 of plasma, kidney, and heart. Group 1: HCC + Gemcitabine. Group 2: HCC + Nanoparticles. Group 3: Normal + Gemcitabine. Group 4: Normal + Nanoparticles. The value represents average ± SD (n = 6). HCC: hepatocellular carcinoma.

Figure 5 compares the percentage of gemcitabine in plasma as well as in liver, kidney, and heart at 60 min after administration of drug/nanoparticles in various treatments. It is apparent from Figure 5 that the greatest percentage of drug disappeared from plasma is in liver tissues, with relatively low amounts being extracted in the kidney and heart. A significantly higher amount of gemcitabine (approximately 64% of administered dose) accumulated in liver tissues of HCC rats (group 2), while it was low in plasma (approximately 12%) at 60 min after administration. Indeed, the high uptake of nanoparticles mainly in liver tissues demonstrate the prospective of the G4 to selectively and effectively target the hepatocytes in HCC rats. Therefore, the plausible justification for the higher gemcitabine deposition in liver tissues might be due to the presence of galactose (asialoglycoprotein receptor ligands) on the nanoparticle surface, which might have provided specific and effective targeting to this organ. This observation is also in agreement with earlier reports wherein the drug-loaded galactosylated chitosan nanoparticles successfully target the asialoglycoprotein receptor, which is overexpressed in HCC [23,24,25]. Reports also indicate that these galactosylated chitosan nanoparticles can enter cells through asialoglycoprotein receptor-mediated endocytosis, and show excellent efficacy in reducing tumor sizes in animal models [23,24,25]. Moreover, the sustained and prolonged release data corroborate the therapeutic efficacy. Therefore, the nanoparticles accumulated in liver tissues may remain there over an extended period and help to maintain a high gemcitabine concentration for a longer time (considering the short half-life) at the diseased site. This prolonged release of gemcitabine in the target site could be an additional advantage, as the nanoparticles will extend the anticancer effect with single administration, which eventually reduces the required dosing frequency and improves patient compliance. In contrast, other organs such as kidney (3.55 ± 0.9%) and heart (0.17 ± 0.11%) had significantly low percentages (*p* < 0.0001) of gemcitabine compared to liver, which is advantageous as it can reduce systemic toxicity and other complications. In group 1, the percentage of gemcitabine accumulated in liver (6.02% ± 0.95%), kidney (4.81% ± 0.90%) and heart (0.23% ± 0.08%) was low, as seen in Figure 5. In the case of groups 3 and 4, the data were relatively similar and the difference was not statistically significant. Overall, the data demonstrate a rapid plasma clearance and higher accumulation of nanoparticles in the HCC tissues, which is suitable for effective treatment of liver cancer.

### 2.8. HCC Measurements.

**Figure 6:**
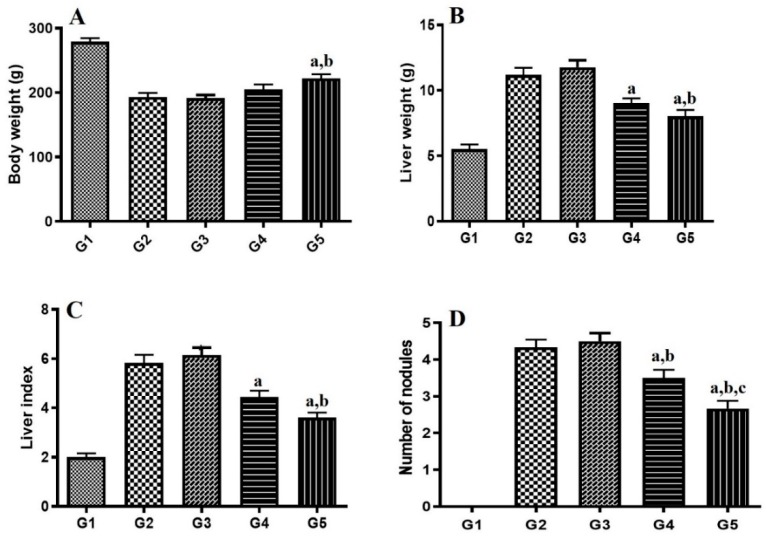
Effect of gemcitabine either alone or formulated in nanoparticles (G4) on (**A**) body weight, (**B**) liver weight, (**C**) liver index, and (**D**) number of nodules. Significantly different from G2 (a), G3 (b) and G4 (c) at *p* < 0.05. G1: Normal control group. G2: Hepatocellular carcinoma (HCC) group. G3: HCC + Placebo. G4: HCC + Gemcitabine. G5: HCC + Nanoparticles. The value represents average ± SD (*n* = 6). Liver index = Liver weight of rat/Bodyweight of rat × 100.

The final phase of the study evaluated the ability of G4 to provide selective liver targeting and consequent improvement of the therapeutic efficacy of gemcitabine in HCC. The effect of gemcitabine either alone or in nanoparticles (G4) on body weight, liver weight, liver index [(liver weight of rat/bodyweight of rat) ×100] and number of nodules (counted on liver surface) is shown in Figure 6. In the present study, induction of HCC has shown significant reduction in body weight during the entire induction phase, which demonstrates that induction reduces food consumption and/or increases metabolic process. Treatment with G4 showed significant improvement in weight loss produced by disease induction as evidenced in Figure 6A. However, gemcitabine alone does not have a significant effect on body weight, which confirms that G4 has a better protective effect compared to the drug alone.

Many studies have suggested that as the tumor progresses, liver weight increases [37]. In the current experiment, the disease control group showed a significant increase in liver weight, as seen in Figure 6B. The groups treated with gemcitabine alone or G4 showed a preventive effect by decreasing liver weight. Liver index is related to aggressiveness of HCC and is an important prognostic marker in HCC patients [38]. It is evident from Figure 6C that liver index is remarkably increased in the disease control group compared to the normal control group, which was found to be decreased significantly by gemcitabine alone (*p* < 0.05) and G4 (*p* < 0.001). In HCC, liver hypertrophy and liver fibrosis lead to development of nodules [39]. The disease control group showed a greater number of nodules, whereas the number of nodules was found to be reduced significantly by gemcitabine alone (*p* < 0.05) and in formulation (*p* < 0.001), as seen in Figure 6D. Overall, the results demonstrated that G4 has an impressive protective effect against HCC.

**Figure 7:**
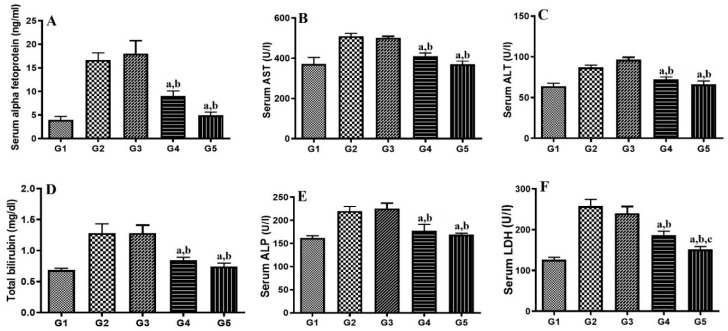
Effect of gemcitabine either alone or nanoparticles (G4) on (**A**) Serum alpha-fetoprotein, (**B**) aspartate aminotransferase (AST), (**C**) alanine aminotransferase (ALT), (**D**) bilirubin, (**E**) alkaline phosphatase (ALP), and (**F**) lactate dehydrogenase (LDH). Significantly different from G2 (a), G3 (b) and G4 (c) at *p* < 0.05. G1: Normal control group. G2: Hepatocellular carcinoma (HCC) group. G3: HCC + Placebo. G4: HCC + Gemcitabine. G5: HCC + Nanoparticles. The value represents average ± SD (*n* = 6).

The diethylnitrosamine-induced HCC model presents clinical features of HCC. Alpha fetoprotein (AFP), an oncofetal serum protein, is the specific liver tumor marker found to be increased in hepatocarcinogenesis [40]. The results in Figure 7A show that the level of AFP is increased in the disease control group, whereas a significant decrease in AFP level was found in the gemcitabine alone (*p* < 0.05) and G4 (*p* < 0.001)-treated groups, which established the better anticancer activity of G4. In HCC, tissue necrosis occurs, which leads to leakage of various liver enzymes into the blood stream due to ruptures of hepatocytes. It was found that serum biochemical liver function markers (AST, ALT, bilirubin, ALP, and LDH) from the HCC-treated group were significantly higher than in the normal control group, as seen in Figure 7. These levels were profoundly decreased by gemcitabine alone and G4, which showed protective roles in HCC. Further, the effect produced by G4 was significantly higher (*p* < 0.05) compared to the drug alone. Thus, treatment showed a protective effect against tissue necrosis, thus inhibiting leakage of these markers into the serum.

**Figure 8:**
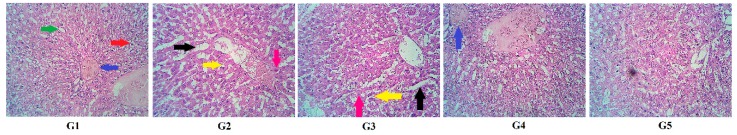
Effect of various treatments on rat liver histopathology. G1: Normal control group. G2: Hepatocellular carcinoma (HCC) group. G3: HCC+Placebo. G4: HCC+Gemcitabine. G5: HCC+Nanoparticles. Sinusoids (green arrow), central vein (blue arrow), radiating hepatocytes (red arrow), dilated sinusoids (black arrow), vacuolated hepatocytes (yellow arrow) and atypical nucleus (pink arrow).

Previous studies have indicated that chronic inflammation and fibrosis are characteristic features of rat HCC [41]. On histopathological examination, the liver histology of normal control rats has no sign of inflammation, fibrosis, or damage in hepatic cells. In the disease (G2) and placebo control (G3) groups, vacuolated hepatocytes, atypical nucleus, and dilated liver sinusoids were noticed, as seen in Figure 8. At the end of the experimental period, both the gemcitabine alone and G4-treated groups showed protective effects on liver architecture with little cellular damage. Both treatment groups also retained cell structure integrity.

**Figure 9:**
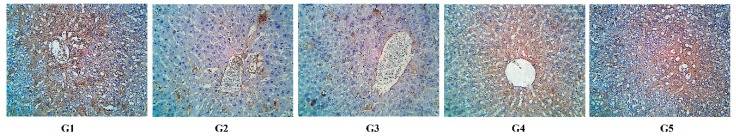
Effect of various treatments on liver immunohistochemistry of p53. G1: Normal control group. G2: Hepatocellular carcinoma (HCC) group. G3: HCC+Placebo. G4: HCC+Gemcitabine. G5: HCC+Nanoparticles. Significantly different from G2 (a) and G3 (b) at *p* < 0.05.

Upon immunohistochemical analysis of the tumor suppressor gene p53, it was observed that the disease control groups (G3 and G4) showed lower expression of p53, which is represented by weak brown stain intensity (borderline to weak) in cells. However, there was strong staining intensity (moderate to strong) found in both the gemcitabine alone and G4-treated groups, which indicates increased expression of p53 after treatment, as seen in Figure 9. Thus, the current study revealed that the inhibitory effect of treatment on HCC is associated with overexpression of p53, which may induce apoptosis of HCC [42,43].

## 3. Materials and Methods.

### 3.1. Materials

Gemcitabine hydrochloride was provided as a gift from Intas Pharm, Ahmedabad, India. Chitosan, lactobionic acid, ethidium bromide, 1-ethyl-3-(3-dimethyl aminopropyl) carbodiimide, *N*-hydroxylsuccinimide, *N,N,N′,N*′-tetramethylethylenediamine, and *N*-nitrosodiethylamine were purchased from Sigma-Aldrich (St. Louis, MO, USA). Sodium Tri-polyphosphate (CDH Mumbai, India) and acetonitrile (Merck Mumbai, India) were purchased commercially. Diagnostic kits for alpha-fetoprotein (AFP), aspartate aminotransferase (AST), alanine aminotransferase (ALT), bilirubin, alkaline phosphatase (ALP), and lactate dehydrogenase (LDH) were obtained from Accucare-Lab Care Diagnostics Pvt. Ltd., Valsad, India. All other excipients used in the development of formulation were of analytical reagent grade and of exceptional quality.

### 3.2. Analysis of Gemcitabine

Measurement of gemcitabine in different samples was done by high performance liquid chromatography (HPLC) system (LC4000+ Series, Jasco, Tokyo, Japan). A HPLC system consists of LiChrospher 100 C18 column (4.6 × 250 mm, particle size 5 µm) with a 20 µL sample loop connected to UV-Visible detector (MD-4010, Jasco, Tokyo, Japan) and a software for data acquisition (ChromNAV 2.0, Jasco, Tokyo, Japan) was used. Elution of gemcitabine was achieved by a mobile phase consisting of acetonitrile and acetate buffer (97:3% *v/v*; pH 5). The column temperature was maintained at 25 °C, while the flow rate of mobile phase was 1 mL/min. Samples (20 µL) were injected and the detection was done at 280 nm [44]. Linear regression analysis indicates good linearity in the concentration of 25–800 ng/mL (r^2^ = 0.995) and the retention time was estimated as 3.54 min. The LOQ (limit of quantitation) and LOD (limit of detection) of this method are 18.90 ng/mL and 6.23 ng/mL, respectively. The coefficient of variation and the accuracy range were 1.89%–8.25% and −2.67–9.05, respectively.

### 3.3. Synthesis of Galactosylated Chitosan

Galactosylated chitosan was prepared by minor modification of a method described by Cheng et al. [45]. In short, accurately weighed amount (2.3 g) of lactobionic acid was dissolved in 50 mL of *N*,*N*,*N*′,*N*′-tetramethylethylenediamine/HCl buffer solution (pH 4.5), which was actuated with 1-ethyl-3-(3-dimethyl aminopropyl) carbodiimide (0.6 g) and *N*-hydroxylsuccinimide (0.14 g). Afterwards, chitosan (2.2 g) was included into the solution at an identical molar ratio to lactobionic acid. The reaction was continued at room temperature (25 ± 1 °C) for 72 h. The solution was dialyzed (MWCO 3500) across distilled water for 72 h at 25 °C and subsequently freeze dried by a lyophilizer (VaCo 2, ZiRBUS, Harz, Germany) to get galoctosylated chitosan. Fourier transform infrared (FTIR) spectroscopic (8400S, Shimadzu, Tokyo, Japan) technique was employed to elucidate the chemical structure of the prepared polymer.

### 3.4. Preparation of Gemcitabine Nanoparticles

Gemcitabine nanoparticles were formulated using ionic gelation method with minor modification of the method described before [46]. Briefly, in a 50 mL beaker, galactosylated chitosan was dissolved in 2% (*v/v*) glacial acetic acid and its pH was adjusted to 5.5 with NaOH. Gemcitabine dissolved in water was added gradually to this solution with continuous stirring, followed by dropwise addition of 1 mL of aqueous sodium tripolyphosphate solution (2% *w/v*) using a syringe with an 18G–1.2 mm needle at rate of 1 mL/min. The solution was further stirred by placing in a magnetic stirrer for 60 min at a speed of 1200 rpm at 25 °C. After incubation, the solution (30 mL) was centrifuged (R-83; Remi, Mumbai, India) twice at 13,000 rpm for 20 min to remove excess amounts of sodium tripolyphosphate and unentrapped gemcitabine. Nanoparticles obtained were washed with deionized water. The product was again dispersed in deionized water, prefrozen at −80 °C (24 h) using lab freezer and freeze dried (24 h) by a lyophilizer (VaCo 2, ZiRBUS, Harz, Germany) using mannitol (5%) as cryoprotectant. Five different formulations (G1–G5) were prepared with varying weight proportions of drug to polymer (1:2 to 1:10).

### 3.5. Characterization of Gemcitabine Nanoparticles

#### 3.5.1. Percentage Yield

The prepared nanoparticles percentage yield was computed applying the following equation:

(1) $$\%\mathrm{Yield}=\frac{\mathrm{Practical}\;\mathrm{yield}}{\mathrm{Theoretical}\;\mathrm{yield}}\times 100$$

#### 3.5.2. Drug Entrapment Efficiency (EE) and Drug Loading (DL)

EE of developed nanoparticles were calculated by the equation:

(2) $$\%\;\mathrm{EE}=\frac{\mathrm{Drug}\;\mathrm{content}\;\mathrm{in}\;\mathrm{nanoparticles}}{\mathrm{Initial}\;\mathrm{quantity}\;\mathrm{of}\;\mathrm{drug}\;\mathrm{included}}\times 100$$

Percentage of DL was estimated [47] as:

(3) $$\%\mathrm{DL}=\mathrm{Quantity}\;\mathrm{of}\;\mathrm{drug}-\frac{\mathrm{loaded}\;\mathrm{in}\;\mathrm{nanoparticles}}{\mathrm{Total}\;\mathrm{quantity}\;\mathrm{of}\;\mathrm{polymer}\;\mathrm{plus}\;\mathrm{drug}\;\mathrm{included}}\times 100$$

#### 3.5.3. Drug Content

Accurately weighed quantity of nanoparticles were allowed to dissolve in glacial acetic acid (1% *v/v*), diluted with mobile phase consisting of acetonitrile: acetate buffer (97:3% *v/v*). The solution obtained was centrifuged and the supernatant layer was decanted and subsequently filtered using 0.2 µm Millex syringe-driven membrane unit (Millipore Corporation, Bedford, MA, USA). Gemcitabine content in the nanoparticles was analyzed by the HPLC methodology described before.

#### 3.5.4. Transmission Electron Microscopy (TEM)

TEM (Tecnai 20 S-twin, Philips, Netherlands) was utilized to observe the properties of nanoparticles, such as particle size, shape, and distribution. Aqueous dispersion of the samples was prepared and a few drops of polymeric dispersion were layered onto a carbon-coated copper stained grid surface of 300 mesh. For obtaining high contrast images, negative staining with phosphotungstic acid (2% *w/v*) was used. Excess samples were wiped with filter paper and the films were air dried at 25 ± 1 °C. The films were inspected at an accelerating voltage of 80 kV and pictures were taken.

#### 3.5.5. Particle Size Characterization and Zeta Potential

The particle size distribution and zeta potential of nanoparticles were evaluated by dynamic light scattering in a Zetasizer (Nanopartica SZ-100, Horiba, Kyoto, Japan) at 25 °C. Nanoparticles were dispersed in non-dissolving fluid, deionized water using ultrasonicator (Transonic T460/H, Elma, Germany) for 1 min before analysis to maintain the particles in suspension and to minimize the aggregation among the nanoparticles.

#### 3.5.6. FTIR Spectroscopy

Spectra of gemcitabine, galactosylated chitosan, the physical mixture, and gemcitabine nanoparticles were obtained by an FTIR spectrometer (Jasco, 6100, Tokyo, Japan). An intimate mixture of samples with KBr (1:5) were prepared by grinding in a mortar and the pellets were made by compression technique employing a hydraulic press (5000–10,000 psi). The pellets were scanned between 400 to 4000 cm^−1^ and the FTIR spectra were recorded.

#### 3.5.7. Differential Scanning Calorimetry (DSC)

The thermal characteristics of gemcitabine, placebo nanoparticles, and gemcitabine nanoparticles were done by DSC (DSC 60, Shimadzu, Japan). Accurately weighed quantities of samples (5 mg) were taken in an aluminum crimped pan and sealed in air-tight conditions. A blank aluminum pan was used as a reference sample during the thermal analysis. DSC thermograms were reported at a heating rate of 10 °C/min in an inert nitrogen atmosphere set at 10–400 °C.

### 3.6. In Vitro Drug Release and Kinetics

The release of gemcitabine from nanoparticles or pure drug was determined by dialysis bag diffusion technique. To 50 mg of nanoparticles or gemcitabine, 2 mL of phosphate-buffered saline (PBS) was added and transferred into a dialysis tube (molecular cut off 12,000–14,000, Spectrum Laboratories Inc., Berkeley, CA, USA). The dialysis tube was immersed in the release medium contains 200 mL of PBS (pH 7.4) set at 37 ± 0.5 °C, which was stirred using magnetic stirrer (200 rpm). Aliquots volume of the sample were collected at specified time periods and separately filtered using 0.2 µm membrane filter. The released amount of gemcitabine was analyzed by the HPLC. A graph was plotted between the cumulative percentage of gemcitabine released against the time and release kinetics were computed applying different mathematic models using below equations [48].

- Zero order model *Q* = *Q*_0_ + *kt*
- First order model *Q* = *Q*_0_ × e*^kt^*
- Higuchi model *Q* = *k* × *t*^0.5^
- Korsmeyer–Peppas model *Q* = *k* × *t^n^*
- Weibull model *Q* = 1 − exp[−(*t*)*^b^*^/*a*^]

where *Q* denotes amount of gemcitabine released in time *t*, *Q*_0_ denotes value of *Q* at zero time, *k* denotes the rate constant, *n* denotes the diffusional exponent, *a* denotes the time constant, and *b* represents the shape parameter. 

### 3.7. Stability

The stability of G4 was determined for three months by storing in a sealed glass vial loaded in a desiccator at room temperature (25 ± 1 °C) and 75% ± 5% relative humidity [49]. Nanoparticles were evaluated visually for physical stability, particle size, drug content (chemical stability), and drug release.

### 3.8. Blood Disappearance and Organ Distribution

Male Wistar rats, weighing between 180–220 g, were obtained from the animal house of Nirma University. The animals were housed under controlled environmental conditions (12 h light/dark cycle and 22–24 °C temperature) and fed with a commercial pellet diet and water ad libitum. The animals were acclimatized to the laboratory conditions for one week before beginning the experiment (ethical approval number; IP/PCEU/FAC/25/2019/033). The plasma clearance and the organ distribution of gemcitabine either free or in combination with nanoparticles were determined in four groups of rats, each consisting of six rats. Groups 1 and 2 were HCC-induced, while normal rats were used in groups 3 and 4. Drug and nanoparticles in vehicle were intravenously administered into the tail vein. Groups 1 and 3 received gemcitabine (1 g/m^2^), while groups 2 and 4 were administered with gemcitabine nanoparticles (1 g/m^2^). Samples of blood were withdrawn from the retro-orbital plexus and transferred to separate heparinized tubes at predetermined time interval such as 0, 5, 10, 20, 30, and 60 min postdosing, respectively. To each sample, the same volume of acetonitrile and 2-propanol were added to ensure precipitation of proteins, centrifuged (12,000 rpm for 10 min), and the supernatant was filtered using 0.2 µm membrane filter. The concentration of gemcitabine was analyzed using the HPLC. Animals from the different groups were sacrificed after treatment to examine the organ distribution of gemcitabine. Selected tissues from the organs (liver, kidney, heart) of rats were harvested, thoroughly cleaned using PBS, swabbed with Kleenex, and the weight of each organ was noted. Then the tissue homogenization was done in PBS using a tissue homogenizer. The homogenate samples were ground with similar volume of acetonitrile and 2-propanol, vortexed for 5 min, centrifuged (10,000 rpm for 10 min), and the top layer fluid was filtered through a 0.2 µm membrane. The drug present in all organs was analyzed by HPLC. 

### 3.9. HCC Experimental Protocol

Male Wistar rats (180–220 g) were classified into five groups, with each group containing six rats (ethical approval number; IP/PCEU/FAC/25/2019/033). The first group, treated as the normal control group, was administered with 2 mL sterile saline. HCC was induced in groups 2–5 via intraperitoneal administration of diethylnitrosamine at a dose of 200 mg/kg body weight. Two weeks later, carcinogenesis was induced with dietary 2-acetylaminofluorene (0.02% *w/w*), which continued for a period of two weeks [50]. Treatment was administered for three weeks by administering the dose into the tail vein three times per week. Group 2 received sterile saline. Group 3 was administered with placebo nanoparticles. Group 4 received gemcitabine (1 g/m^2^), while group 5 was administered with gemcitabine nanoparticles (1 g/m^2^). The dose of gemcitabine was determined as per the equation described in the literature [51]. After the completion of the investigation, rats were weighed and blood samples were drawn and centrifuged (4000 × *g* for 15 min) to separate the serum that was used for biochemical analysis of the tumor marker AFT and liver function tests of AST, ALT, bilirubin, ALP, and LDH. Later, the animals were sacrificed, and the liver was excised, immediately weighed (to determine liver index), and liver nodules were counted. Liver slices were taken and fixed in 10% neutral buffered formalin for histopathological and immunohistochemical evaluations. Liver specimens were dehydrated by immersing in increasing concentration of alcohol, cleared in xylene, embedded in paraffin, sectioned (~5 µm) using microtome and stained with hematoxylin–eosin and p53 antibody for histopathological and immunohistochemical examination, respectively. Histopathological sections were microscopically examined and scored. The immunohistochemical staining of p53 was assessed according to the immune-reactive score (IRS). The staining intensity was scored manually and graded as follows: 0, no staining; 0.5, borderline staining; 1, weak staining; 2, moderate staining; 3, strong staining.

### 3.10. Statistical Analysis

The data were analyzed by Student’s *t*-test or one-way ANOVA using GraphPad Prism software (San Diego, CA, USA). *p* value less than 0.05 is considered statistically significant.

## 4. Conclusions

Our gemcitabine-loaded galactosylated chitosan nanoparticle formulation was successfully designed and developed by ionic gelation. The narrow size distribution of the nanoparticles with high drug content suggested that the developed formulation is favorable for in vivo administration and subsequent liver targeting. FTIR studies confirmed the absence of drug–polymer interaction, and DSC thermograms indicate the amorphous state of gemcitabine present in the nanoparticles. The sustained and prolonged release pattern of gemcitabine displayed by the nanoparticles is beneficial as this might provide gradual and prolonged delivery of gemcitabine in the liver. The clearance of gemcitabine in the plasma was rapid from nanoparticles as compared to pure drug in HCC rats. Organ distribution data demonstrated excellent liver targeting by G4, which is likely to enhance the gemcitabine therapy and can eventually lead to active cancer inhibition. Biochemical, histopathological, and immunohistochemical results suggest a significant antitumor effect of G4 in rat HCC. Therefore, the selective and preferential delivery of gemcitabine to liver cancer tissues could be a feasible alternative therapeutic approach in the treatment of HCC.

## Figures and Tables

**Table 1:** Effect of formulation parameters on physicochemical characteristics of gemcitabine-loaded galactosylated chitosan nanoparticles.

F	Drug: Polymer Ratio	% Yield	% EE	% Drug Loading	% Drug Content	Mean Diameter (nm)	Zeta Potential (mV)
**G1**	1:2	68.5 ± 6.1	54.6 ± 3.2	18.2 ± 2.9	93.5 ± 2.5	156.8 ± 30.1	19.3 ± 2.6
**G2**	1:4	72.5 ± 7.8	64.2 ± 5.9	12.8 ± 1.6	94.1 ± 2.1	157.2 ± 38.3	21.5 ± 3.1
**G3**	1:6	73.4 ± 5.9	66.4 ± 6.1	9.5 ± 2.1	94.6 ± 2.8	162.0 ± 34.2	22.3 ± 2.5
**G4**	1:8	74.2 ± 7.4	72.1 ± 6.5	8.0 ± 2.3	95.9 ± 3.2	164.1 ± 39.5	20.4 ± 3.9
**G5**	1:10	73.2 ± 6.5	71.9 ± 4.2	6.5 ± 2.2	94.4 ± 2.8	171.3 ± 35.2	21.8 ± 2.1

All values are expressed as mean ± S.D; *n* = 6. F: Formulation; EE: Entrapment efficiency.
